# Linking Climate to Incidence of Zoonotic Cutaneous Leishmaniasis (*L.* *major*) in Pre-Saharan North Africa

**DOI:** 10.3390/ijerph10083172

**Published:** 2013-07-31

**Authors:** Lahouari Bounoua, Kholoud Kahime, Leila Houti, Tara Blakey, Kristie L. Ebi, Ping Zhang, Marc L. Imhoff, Kurtis J. Thome, Claire Dudek, Salah A. Sahabi, Mohammed Messouli, Baghdad Makhlouf, Abderahmane El. Laamrani, Ali Boumezzough

**Affiliations:** 1 Hydrospheric and Biospheric Sciences Laboratory, NASA’s Goddard Space Flight Center, Maryland, MD 20771, USA; E-Mails: ping.zhang@nasa.gov (P.Z.); kurtis.thome@nasa.gov (K.J.T.); 2 Laboratory of Ecology & Environment, Cadi Ayyad University, Marrakech 40000, Morocco; E-Mails: kahimkholoud@gmail.com (K.K.); aboumezzough@gmail.com (A.B.); 3 Faculty of Medicine, Sidi Bel Abbes 22000, Algeria; E-Mail: leilahouti@yahoo.fr; 4 Florida International University, Florida, FL 33199, USA; E-Mail: tblakey@fiu.edu; 5 Department of Medicine, Stanford University, Stanford, CA 94305 USA; E-Mail: krisebi@essllc.org; 6 Earth Resources Technology Inc., Laurel, MD 20707, USA; 7 Joint Global Change Research Institute at the University of Maryland, MD 20740, USA; E-Mail: marc.imhoff@pnnl.gov; 8 Bethesda-Chevy Chase High School, MD 20814, USA; E-Mail: cdudek@smith.edu; 9 Hydrometeorological Institute of Training and Research, Oran 31025, Algeria; E-Mail: salah_sahabi@yahoo.com; 10 Laboratory of Hydrobiology, Ecotoxicology and Sanitation, Cadi Ayyad University, Marrakech 40000, Morocco; E-Mail: messouli@gmail.com; 11 Establishment of Local Public Health, Saida 20000, Algeria; E-Mail: makhloufbaghdad@yahoo.fr; 12 Center of Epidemiology and disease Control, Ministry of Health, Rabat 10010, Morocco; E-Mail: laamrani55@gmail.com

**Keywords:** cutaneous leishmaniasis, surface climate indicators, incidence, climate, NDVI, North Africa

## Abstract

Shifts in surface climate may have changed the dynamic of zoonotic cutaneous leishmaniasis (ZCL) in the pre-Saharan zones of North Africa. Caused by *Leishmania major*, this form multiplies in the body of rodents serving as reservoirs of the disease. The parasite is then transmitted to human hosts by the bite of a Phlebotomine sand fly (Diptera: Psychodidae) that was previously fed by biting an infected reservoir. We examine the seasonal and interannual dynamics of the incidence of this ZCL as a function of surface climate indicators in two regions covering a large area of the semi-arid Pre-Saharan North Africa. Results suggest that in this area, changes in climate may have initiated a trophic cascade that resulted in an increase in ZCL incidence. We find the correlation between the rainy season precipitation and the same year Normalized Difference Vegetation Index (NDVI) to be strong for both regions while the number of cases of ZCL incidence lags the precipitation and NDVI by 2 years. The zoonotic cutaneous leishmaniasis seasonal dynamic appears to be controlled by minimum temperatures and presents a 2-month lag between the reported infection date and the presumed date when the infection actually occurred. The decadal increase in the number of ZCL occurrence in the region suggests that changes in climate increased minimum temperatures sufficiently and created conditions suitable for endemicity that did not previously exist. We also find that temperatures above a critical range suppress ZCL incidence by limiting the vector’s reproductive activity.

## 1. Introduction

Leishmaniases are among the most important emerging and resurging vector-borne diseases, second only to malaria in terms of the number of affected people. Leishmaniases are endemic in 98 countries and three territories worldwide and threaten about 350 million people. It is estimated that 14 million people are infected worldwide with about two million new cases occurring each year. The disease contributes significantly to the spread of poverty, because of its expensive treatment, and imposes a heavy economic burden, including loss of income [1].

Among all leishmaniases, cutaneous leishmaniasis (CL) is the most common. There are about 214,000 cases reported each year and the estimated annual CL incidence ranges from 691,000 to 1.2 million cases (90% in Afghanistan, Algeria, Saudi Arabia, Brazil, Peru, Iran and Sudan) [1,2]. The Middle East and North Africa region harbor around 15% of the global leishmaniasis burden exclusively attributable to CL [3], while the disease poses an increasingly serious public health problem in the Maghreb region [4].

The incidence of CL has increased across the globe and urbanization is indicated as a key factor in this increase [5]. Observations show that CL has expanded beyond its natural ecoregion, especially in the arid regions of the Middle East and North Africa where significant environmental changes occurred. References [2], [6] and [7] highlight the spread of CL from the Algerian arid zones northward towards the semi-arid areas and suggest that climate change and desertification observed in the steppe of the northern Sahara could have played a role in this territorial expansion of the disease. Similarly, in Morocco, CL is spreading at a fast rate, from the Atlantic coast south of the Anti-Atlas to the Northeastern regions passing through the pre-Saharan zones south of the Atlas Mountain, in particular in the Souss-Massa-Draa valley and the province of Errachidia and spreading east into the Algerian territory [1,2,8].

Cutaneous leishmaniasis (CL) is caused by a protozoan parasite of the genus *Leishmania*, which multiplies in the body of rodents serving as reservoirs of the disease. The parasite is transmitted to human hosts by the bite of a vector that was previously fed by biting an infected reservoir. There are at least three *Leishmania* species that can cause CL [1]; *L. infantum*, *L. tropica* and *L. major*. For each of these forms, the reservoir and the vector vary from place to place. In Algeria, a country with the highest number of reported CL cases per year in the Mediterranean region between 2004 and 2008 [2], zoonotic cutaneous leishmaniasis (ZCL) caused by *L. major* is dominant and distributed over a wide band across the southern arid zones with about 10,000 cases recorded annually [2,7,9]. Cases of CL caused by *L. tropica* and *L. infantum* have also been isolated, but are less frequent [1] and are confined to the north of the country, geographically separated from *L. major* CL by the Tell Mountains which constitute a natural barrier [6]. Recently, however, foci of CL have emerged north of the Tell Mountains with identification of the parasites showing that all strains belonged to *L.* *major* MON-25 and investigations into the reservoirs pointed to the (fat) sand rat (*Psammomys obesus*) and Shaw’s jird (*Meriones shawi*) as proven hosts [6]. The first isoenzymatic characterization of the *Leishmania* strains responsible for cutaneous leishmaniasis in Algeria was recently presented [7]. The studyperformed in the northeastern part of Algeria analyzed 16 samples taken from a large pool of 259 infected persons using isoenzyme analysis. Out of the 16 strains, the isoenzymatic identification showed the presence of three Leishmania species: eight were *L. major* (50%), seven were *L. infantum* (44%), one was *L. killicki* (6%) and none of the strains were *L. tropica*. Like in Algeria, in Morocco, CL is caused by the same *Leishmania* species [1]. Its geographical distribution covers large parts of the country with *L. infantum* in the northern regions and *L. tropica* in the central to western semi-arid regions. However, the ZCL caused by *L. major* is exclusively found in the Saharan regions in the south and the south-east with outbreaks moving in waves from west to east and no evidence of overlap in the region of Errachidia [10,11]. The reservoir in populated areas is *Meriones shawi*, but it is suggested that, as in Algeria, there is a ‘sylvatic’ reservoir system that ‘feeds’ this urban system, with *Psammomys obesus* as reservoir and the sandfly *Phlebotomus papatasi* as the main vector [9].

No or limited isoenzymatic or DNA strain characterization has been carried out to identify the CL causative agent in these regions. However, the data selected for this study was based on lesions having a form and seasonality consistent with L. major CL, although other species may have been present. In this study we are specifically interested in the ZCL, caused by *Leishmania* *major*, and variation in its seasonal and interannual pattern as influenced by surface climate variables. This clinical form is transmitted by the sandfly (*Phlebotomus* *papatasi* Scopoli) vector with *Psammomys obesus* and *Meriones shawi* serving as reservoirs. As for other vector-borne diseases, important determinants of this ZCL include the vector activity and reproductive periods. These factors control the vector density and biting rate and thus the number of cases of the disease. Pathogens, reservoirs and vectors each survive and reproduce within a range of climatic conditions [1]: temperature and moisture have the most influence, while wind speed is also important [6,8]. The leishmaniasis complex, parasite-reservoir-vector, evolves in specific geographic regions and is sensitive to environmental changes that can affect the parasite, the reservoir and the vector as well as their dynamic interaction and territorial extension.

There is evidence that changes in climate contribute significantly to the increase in the number of cases and expansion of the range of ZCL [12,13,14,15]. As with other vector-borne diseases, seasonal patterns in the number of cases and vector abundance suggest that ZCL transmission is sensitive to the physical environment [16]. Seasonal patterns have been widely documented in [17,18,19,20] while the correlation between vector density and the number of case is described in [19,20]. More specifically, seasonal variations of climate indicators such as minimum and maximum temperatures as well as rainfall amount and length of the rainy season affect the physiological behavior of the leishmaniasis complex producing thus seasonal patterns in the incidence of ZCL [21]. Weather and climate variables play an important role in ZCL incidence as they can constrain or exacerbate favorable conditions for the disease such as an acceleration of the development of the parasite or synergistic changes in reservoir and vector populations that cause an explosion in the vector population. For example, increase in precipitation may increase the vegetation density and thus the number and quality of breeding sites for both the rodents and the sandflies [22].

Changes in climate, although small, may also have an impact on the geographical distribution of ZCL reservoirs and vectors and on their density, their activity and their reproductive periods (e.g., [15]). For example, increases in minimum temperature and humidity will shorten the incubation period (time required for development of the infectious agent in the body of the vector) and shorten the maturation period of the vector, which could increase its vectorial capacity [23]. On the other hand, persistent warming and drought would diminish this capacity. The strongest effects of climate on the ZCL cycle may happen at the extremities of the optimal activity temperature range, which for the sandfly are in the vicinity of 15–18 °C for the low and 32–40 °C for the high end [23,24]. If ambient temperature reaches the upper values of this range, the transmission could cease completely, seriously reducing the cases of ZCL. Around 30–32 °C, the vectorial capacity is observed to increase significantly due to the shortening of the incubation period, despite a decrease in the vector’s survival [25]. This suggests that the vector’s physiology responds to subtle changes in weather and climate, that changes in the environment could affect the dynamic between the components of the leishmaniasis complex in such ways as to either suppress the disease if the environmental conditions extend outside of the optimum range or create conditions for endemicity if environmental conditions are within the optimum range. 

While there have been several studies of ZCL (e.g., [1,4,5,23,24]) only few (e.g., [6,15]) have described it in pre-Saharan North Africa and analyzed variations in the climate and vegetation variables contributing to its seasonal and interannual pattern. More studies are needed to improve the understanding of the ZCL cycle and the prediction of its evolution. In this study, we describe a functional empirical relationship between indicators of surface climate, including satellite observed vegetation density, and the incidence of ZCL during the 1990–2009 period in two regions, north of the Saharan desert, where ZCL is confirmed, widespread and is expanding northward [6], and a warming of up to 2 °C is expected during the next few decades [26]. 

## 2. Data and Method

**Figure 1:**
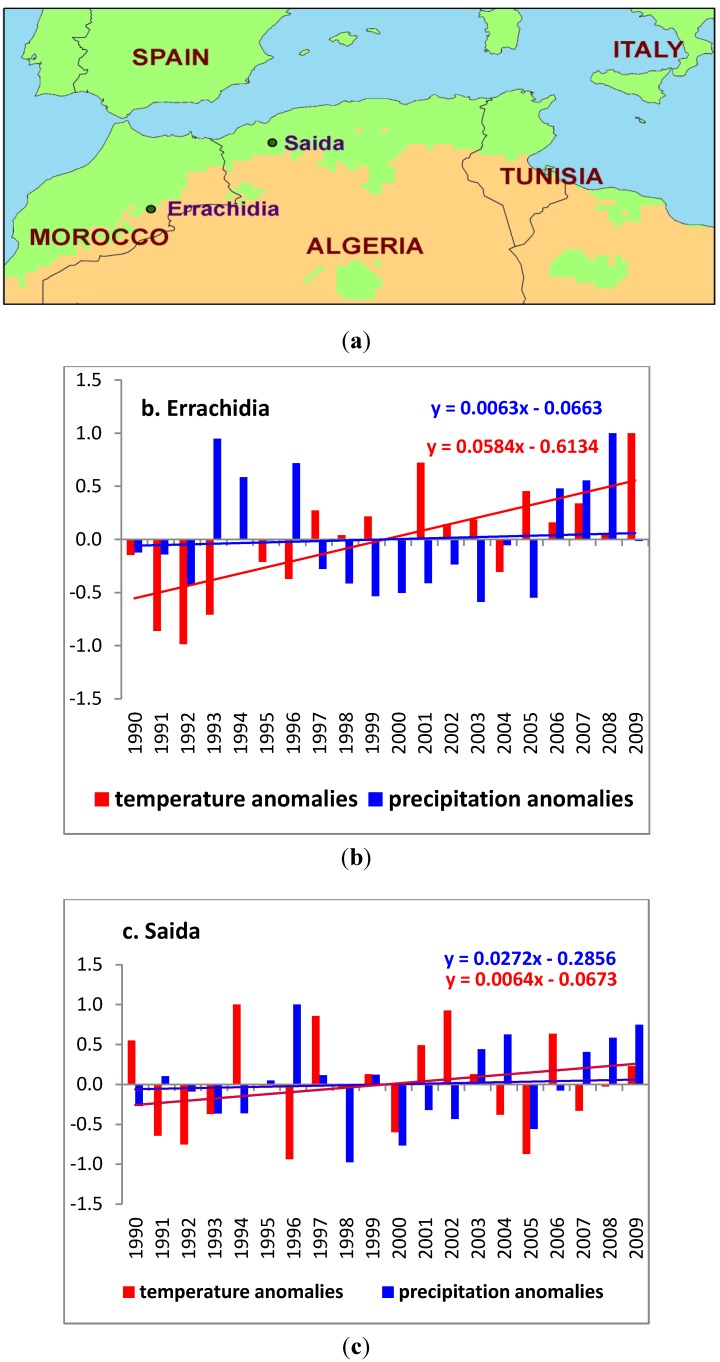
(**a**) Study area, showing the two provinces of Saida and Errachidia; (**b**) Annual mean temperature and precipitation anomalies for Errachidia; (**c**) Annual mean temperature and precipitation anomalies for Saida.

The study area is represented by two provinces (Figure 1(a)), located in northwest Algeria (Province of Saida) and in northeast Morocco (Province of Errachidia) for which concurrent monthly climate and ZCL-cases data were recorded for the period 1990 to 2009. As in the case of Sidi Bouzid (central Tunisia) [15], these two sites are considered as pilot centers for the study of ZCL and have accumulated a fair level of experience in diagnosing it. Most importantly they have a long and reliable data record for this rather data-sparse region [27,28]. This region has experienced a significant increase in the incidence of ZCL [6,10,11] and has also experienced warming and a slight increase in precipitation [29] observable as a trend over the study time period (Figure 1(b,c)).

For Saida, the source of the disease is confined within the focal area of Ain Skhouna, a large wetland surrounded by the semi-arid steppe and home to about 7,500 inhabitants in 2008 [30], whereas for Errachidia the disease is spread all around the province with large concentrations in the central regions of Rissani, Tinjdad and Goulmima. The total population of the province of Errachidia in 2009 was estimated at 600,000 [31]. Both provinces are under the influence of a semi-arid climate regime and are characterized by relatively cold and humid winters and hot and dry summers [32,33].

Over the last 20 years, the annual mean temperature for the large area of the province of Saida was around 18.50 °C with a standard deviation of 0.61 °C and the annual precipitation varied between a minimum of 212.50 mm and a maximum of 538.00 mm around an average annual value of 353.47 ± 96.34 mm. The annual mean temperature for the area surrounding the province of Errachidia was slightly warmer, around 20.62 °C, with a standard deviation of 0.71 °C and the annual total precipitation was 134.28 ± 64.37 mm. Although the region around Errachidia had slightly warmer and drier climatic conditions, it exhibited similar anomalies during the period of analysis (Figure 1(b,c)).

**Figure 2:**
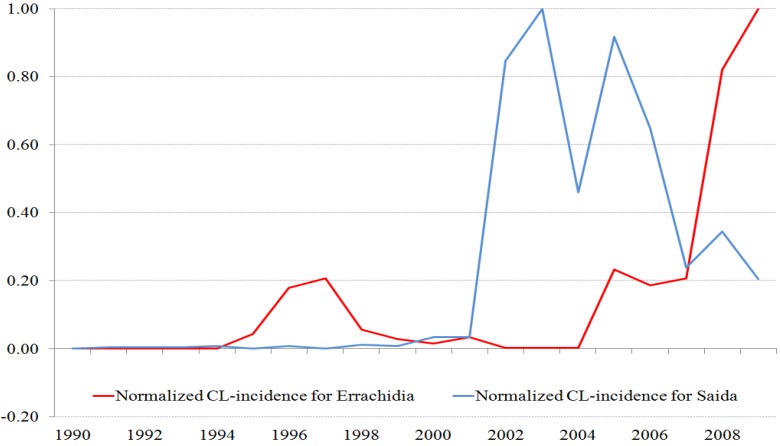
Normalized time series of ZCL-cases for the two provinces. The maximum number of cases for Saida is 267 (population 7,500 inhabitants) and for Errachidia is 1596 (population 600,000 inhabitants).

For Saida, the number of ZCL infections reported each month was provided by the National Institute for Public Health of Algiers—Annual Epidemiologic Data collection (1989 to 2007) and was completed for 2008 and 2009 by data obtained directly from the services of epidemiology and preventive medicine (SEMEP) for the region [34]. For Errachidia data were obtained from the Directorate of Epidemiology and Fight against Diseases (DELM) [35]. In both sites data were collected in health facilities by medical staff using standard protocols relevant to the health department in each country. A case of ZCL is defined as any case reported and/or recorded in health facilities in the two study regions during the common period 1990-2009. Although the appearance of ZCL cases (lesions) can be variable and dependent on strain virulence and host immunity, and CL diagnosis has improved over the years; there was a strong confidence from the staff that collected the data that these effects have minimal impact on the data series obtained from both sites (Figure 2). 

In order to compare the ZCL-seasonal and annual variations in the two provinces, we normalize the time series by their respective maxima. The time stamp of these data sets may be off by one month as cases reported at the beginning and end of the month are not distinguished.

For the two provinces, monthly mean minimum, maximum and mean temperatures as well as mean relative humidity, monthly total rainfall and monthly Normalized Difference Vegetation Index (NDVI) from the Advanced Very High Resolution Radiometer (AVHRR) were obtained and analyzed in terms of their relation to the number of cases of ZCL. The NDVI is an aggregate measure of the vegetation density and health.

Laboratory studies of the sandfly at constant temperature are used to establish annual temporal ranges of the vector’s active period (AP) and reproductive period (RP). To remain as close as possible to laboratory results, we use monthly composite data over the period of analysis.

## 3. Results and Discussion

### ZCL and Climate Variation

Both Saida and Errachidia provinces reported a significant increase in ZCL incidence during the latest decade. Since the first reported case of ZCL in 1991, 1,275 cases were recorded in Saida by the end of 2009, with the vast majority of the cases (99%) reported between 2000 and 2009. Several epidemic peaks were observed, with the highest peak of 267 cases reached in 2003 followed by a significant reduction in 2004 as shown in Figure 2. Similar interannual variation in ZCL-incidence is observed in Errachidia. However, only about 907 cases were reported between 1994 and 2003 and the large majority of the cases (85.5%) occurred between 2004–2009, with large epidemic peaks in 2008 and 2009. Latest reports for Errachidia, not shown, indicate a further increase in 2010 and a relative decrease in ZCL-incidence to 538 cases in 2011. 

Because of the abrupt change in ZCL infection rate occurring around year 2000 for Saida and 2004 for Errachidia, and because the objective of this study was to analyze the relationships between climate variables and disease incidence, the twenty years of climate data were separated into two sub-periods: P1 representing the decade with low incidence rates and P2 representing the period with high incidence rates. For the province of Saida, these periods correspond to 1990–1999 and 2000–2009, respectively whereas for the province of Errachidia they correspond to 1994–2003 and 2004–2009, respectively. Because of data availability for Errachidia, the composite analysis of P2 was performed over 6 years. This separation allows us to compare climate indicators during low rates of incidence with those prevailing during the high incidence period, while the decadal scale analysis provides statistical robustness to the results. Both provinces experienced a warming trend over the period of analysis as shown in Table 1 with the Saida province experiencing a stronger warming and Errachidia a larger moistening.

**Table 1:** Comparison of Decadal Climate and Leishmaniasis Data by Study Site.

***Study Site***	**Saida**		**Errachidia**	
**Decade**	**P1 (1990–1999)**	**P2 (2000–2009)**	**P1 (1994–2003)**	**P2 (2004–2009)**
Annual Minimum Temperature (°C)	12.82	13.55	14.03	14.35
Rainy Season Precipitation (mm)	281.04	308.49	85.20	128.90
ZCL occurrence (percent of the total for 1990–2009)	0.94%	99.06%	14.5%	85.5%
Normalized Difference Vegetation Index	0.39	0.42	0.14	0.16

## 4. Trophic Cascade

For the study area, the maximum vegetation density in a typical year occurs from February through April as indicated by monthly composite NDVI values over the 20 year study period. Because the majority of the study area precipitation occurs during the winter, summer vegetative growth is water limited. During winter, however, when water is available, low temperatures limit vegetative growth [32,36]. 

**Figure 3:**
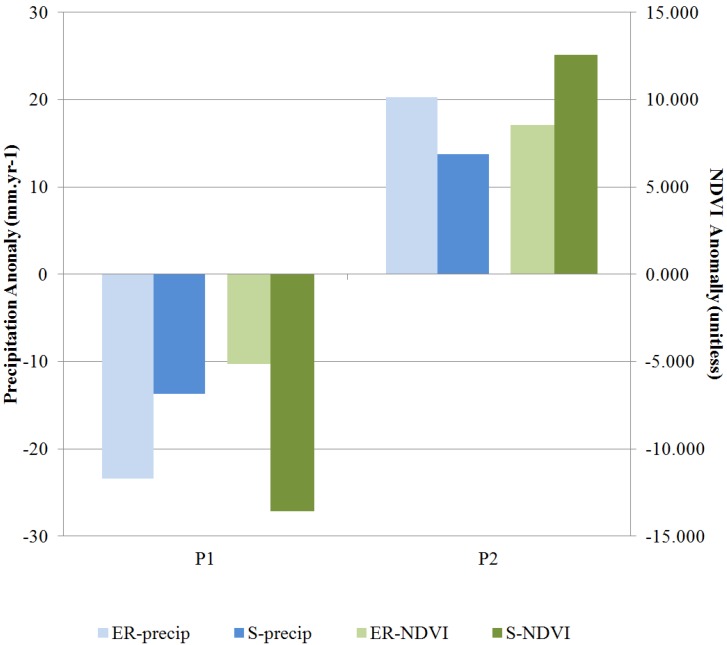
Changes in rainy season precipitation and growing season NDVI from P1 to P2 (see text for details). Changes in NDVI values were scaled by 1,000 for plotting purposes. Prefix ER is for Errachidia and S for Saida.

Figure 3 shows that for Saida P2 received a greater rainfall amount than P1 during the local rainy season defined here as September through April. Accordingly, the vegetation density for the growing season, expressed as the average NDVI value from February to May, was greater in P2 than P1. The same trends in precipitation and NDVI were observed in Errachidia, except with greater strength. In Saida, the precipitation increased by about 5% in P2 and was accompanied by a 3% increase in NDVI. In Errachidia, the positive rainfall anomaly during P2 was about 19% and led to an increase in vegetation density of about 6%. 

Our analysis shows a pattern that confirms the trophic cascade suggested by [22] over the semi-arid area of New Mexico (USA) for Hantavirus, where they found the Fall-Spring precipitations to be correlated to the same year NDVI and to rodents’ density 1 year later. We find a comparable correlation between the precipitation, received during the rainy season, preceding and coincident with the growing season of a particular year, and the trend in NDVI for both Saida (r = 0.68) and Errachidia (r = 0.59). This result is also in line with those of [37] who showed that plant cover responded to precipitation during the same growing season but rodent population lagged at least one year behind in two sites in the desert of North America. In our study region, the typical vegetation is composed essentially of *Atriplex* *halimus* L. (Chenopodiaceae), a widespread perennial drought-resistant C4 shrub [38]. Rodents locate their burrows directly beneath the Chenopod bushes which also constitute their preferred food source [39]. Increasing amounts of vegetation and rodents also promote sandfly activity. Sandflies feed on the juices of plants [3,40] and use rodent’s burrows to survive adverse daytime conditions [24].

Similar to the trophic cascade proposed by Yates *et al*. [22] and Ernest *et al*. [37], we postulate that an increase in precipitation and vegetation density as evidenced in P2 for Saida and Errachidia would support a larger number of local rodents. These two conditions produce a synergy that promotes sandfly density and activity and will ultimately increase the rate of biting and ZCL-infections.

**Figure 4:**
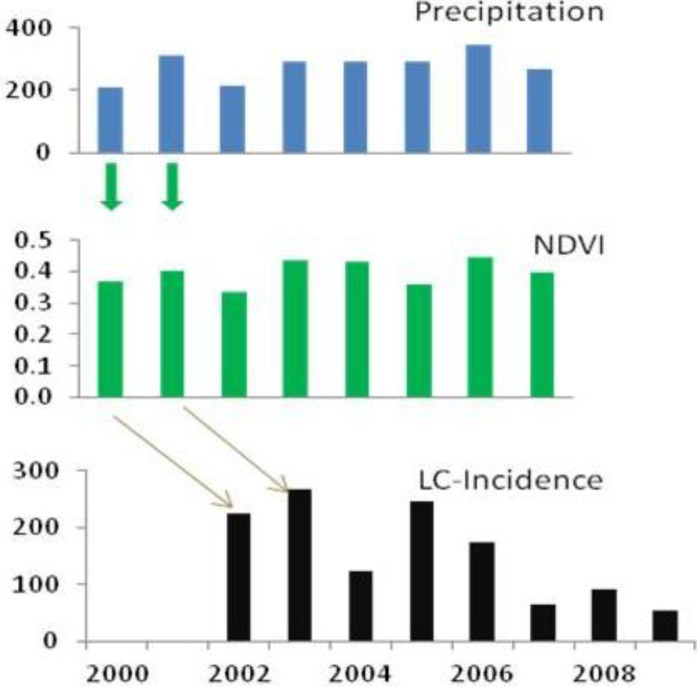
Relationship between precipitation, NDVI, and ZCL-incidence in the province of Saida. Rainy season (September through April) precipitation, growing season (February through May) vegetation density and annual total ZCL-cases. Note the correspondence of pattern dynamics between temporal changes of precipitation and NDVI values. The ZCL-cases lag precipitation and vegetation by two years.

While the correlation between precipitations and growing season NDVI can be observed within the same annual cycle, there are time-lags in the correlation between precipitations and ZCL-incidence (Figure 4). In both provinces, we find that the rate of ZCL-incidence lags the change in precipitation by two years, with stronger correlations for Errachidia than for Saida. For example, in Errachidia the NDVI explains about 50% of the variance of ZCL-incidence two years later and the rainy season precipitation explains about 27%. The NDVI appeared to be a better predictand in Errachidia and rainy season precipitation was a better indicator in Saida. In this analysis we focus more on the pattern of changes rather than the statistical significance of a particular correlation. Although precipitation amount and vegetation density are indicators that better explain the physical relationships in the proposed trophic cascade, we find that they are strongly modulated by extreme temperatures. Indeed ZCL-incidence in a given year is best described by vegetation density two years prior and the previous year maximum and minimum temperatures, indicating that while precipitation and vegetation abundance are necessary conditions for a ZCL-outbreak they could be strongly mitigated or exacerbated by extreme temperatures. This multiple relationship explains more than 70% of the total variance of ZCL-incidence over the region, with a *p*-value of 0.08.

**Figure 5:**
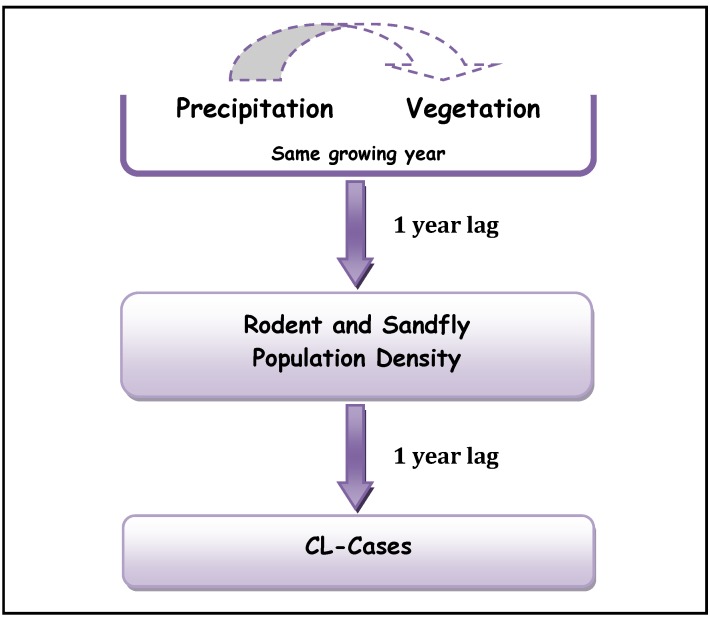
Proposed Trophic Cascade in Leishmaniasis complex.

Ernest *et al.* [37] and Yates *et al.* [22] found that rodent population density displayed a 1-year lag with precipitation. This 1-year lag allowed the rodents to undergo several reproductive cycles. Abundant fall-spring precipitation and spring vegetation enable rodents to continue to increase in population beyond one growing cycle (by winter breeding) and result in maximum rodent population lagging the increases in precipitation and vegetation density. Although no data on rodent’s density was collected during this study and our results and those of Ernest *et al*. [37] and Yates *et al.* [22] are obtained over different regions and for different pathogens, they reached similar conclusions concerning the lags between precipitation, vegetation density and ZCL-incidences. Our analysis suggests an additional 1-year lag between the rodent population density and ZCL-incidence and supports a mechanistic 1-year lag in the cascade between the different trophic levels in the leishmaniasis complex (Figure 5).

### 4.1. Pathogen Cycle and Climate

The annual ZCL cycle was modulated by climate, with extreme temperatures and moisture and their interactions playing important roles, especially during the period of transmission [41,42]. We explored the seasonal variation of ZCL-incidence and its relation to sandfly activity as influenced by surface climate indicators. We consider a rodent population in P2 that was amply supported by the abundance of vegetation and was capable of pathogen transmission year-round. We do not consider the fluctuations of prevalence or incidence peaks based on intrinsic factors such as climate conditions and rodent’s age as described in [43] which suggest other causes of ZCL peaks in central Tunisia associated with the interaction of reservoir and parasite populations. We also do not consider human factors such as immunity development or migration of non-immunes populations to the study regions. We focus on the analysis of the seasonal ZCL cycle based on the impact of climate on the sandfly physiological activity. 

The vector, *P. papatasi*, has been widely studied (e.g., [10,44,45]) and modeling studies have been used to predict its range expansion associated with global warming [46]. The sandfly is known to be nocturnal, with most biting activity occurring at night [24,47]. The life cycle of the vector at constant temperature was observed to require five to eight weeks from conception to death, if conditions did not initiate diapause [23]. During diapause, larval development is suspended once a minimum temperature threshold is reached (similar to hibernation), to ensure that the adult emerges under suitable conditions.

Table 2 defines the vector’s physiologic periods in terms of extreme temperature thresholds. The distribution of the sandfly is not well understood but is known to be highly dependent on environmental conditions [24]. Increases in temperature are likely to be conducive to the development of *Leishmania* organisms and sandfly vectors [47]. It was determined that the vector could not survive outside the temperature range of 10 to 40 °C [24] while reproduction was not possible below 15 °C [23]. These temperature thresholds are used to establish annual ranges of *P.* *papatasi* Active Period (AP) and Reproductive Period (RP) based on observed monthly composite minimum and maximum temperature data over the study region.

**Table 2:** Threshold temperatures (°C) of *P. papatasi* Physiologic Activity.

**Period**	**Minimum Temperature**	**Maximum Temperature**
Active Period (AP)	10	40
Reproductive Period (RP)	15	40

Our climate record shows that during the second decade P2, the average maximum temperature for the province of Saida during the hottest months (July and August) was approximately 36 °C with a standard deviation (SD) of 1.6 °C; while in Errachidia it reached 39.08 °C with SD = 0.86 °C. Although temperature in Errachidia reached high values during July in P2, it may not have directly impacted the sandflies as these nocturnal insects seek refuge during the hottest parts of the day. Minimum temperatures, however, which drop well below the vector’s physiologic thresholds in winters, appear to be more critical in modulating the sandflies’ seasonal cycle over the study area and period.

**Figure 6:**
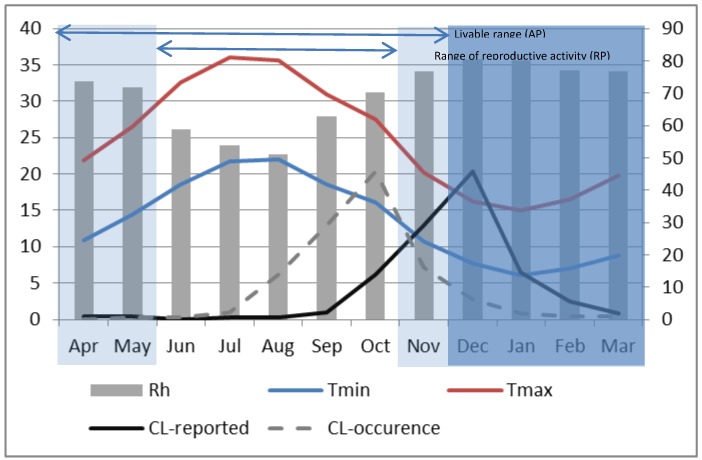
Monthly composite relative humidity (bar, right axis). Maximum (red) and minimum (blue) temperatures (left axis) for P2 at Saida. Dark shading represents the vector’s period of hibernation. Active and Reproductive periods are indicated by horizontal bars at the top of the graph. The dark solid line represents the monthly average reported ZCL-cases during P2 and the dashed line shows the presumed ZCL-occurrence with a 2-month lag (right axis).

Using monthly composite relative humidity, minimum and maximum temperatures rounded to the nearest degree and the thresholds values defined in Table 2, the vector’s physiological periods were identified for both provinces and over decadal time-scales. For example, in Saida during P2 (Figure 6), the AP extends over 8 months from April until November with a hibernation period ranging from December through March when minimum temperatures are observed to drop below the 10 °C threshold. Within the AP, the RP persists for five months from June through October. Although temperature appears to have large effects on the dynamics of *P.* *papatasi*, other environmental factors such as relative humidity, rainfall and photoperiod are also relevant to the life cycle of this species [23]. Except for the month of July and August, the relative humidity was above 60% during the AP with a photoperiod (L:D) varying from 13:11 in April to 10:14 in November. In general, climate data for the region are within the range of livability described by [48] of 27 ± 1 °C, 65 to 75% relative humidity and a 14:10 (L:D) photoperiod.

Transmission of vector-borne diseases such as ZCL is dependent on the duration of the vector’s active period (AP). Where favorable temperatures and other conditions allow for multiple life cycles, a larger sandfly population is created and there is greater probability of contact with infected and non- infected hosts [24]. For the study region and particularly in Saida during P2, the annual peak in sand fly abundance occurred in October (Figure 6), at the end of the reproductive period (RP) where the maximum number of successive generations has contributed to population increase and where weather conditions were optimum: temperatures ranged between 16.04 °C and 27.54 °C, ambient relative humidity was slightly above 70% and the day length was about 11 h. The rationale is that the sandfly population would grow gradually as overwintering larvae emerge and then increase sharply beginning about two months (the time required to go from conception to adult for the female vector was found to be 56–62 days at temperature between 25 °C–28 °C, with the duration increasing as temperature decreases from this range and decreasing for temperatures above this range) into the RP as the population multiplies. It reaches a peak at the end of the RP and then declines sharply at the onset of cold weather when the minimum temperature gets closer to 10 °C in November. Considering that the month with the highest ZCL cases coincides with the month of highest vector abundance [24], our data suggest a 2-month lag between the reported infection date and the presumed date when the infection actually occurred as shown in Figure 6. Although reported monthly ZCL-incidence data carry an uncertainty of about 30 days, this result is still within the range of incubation periods in humans estimated between 8 and 12 weeks in central Afghanistan [21] and in Algeria [34]. 

**Figure 7:**
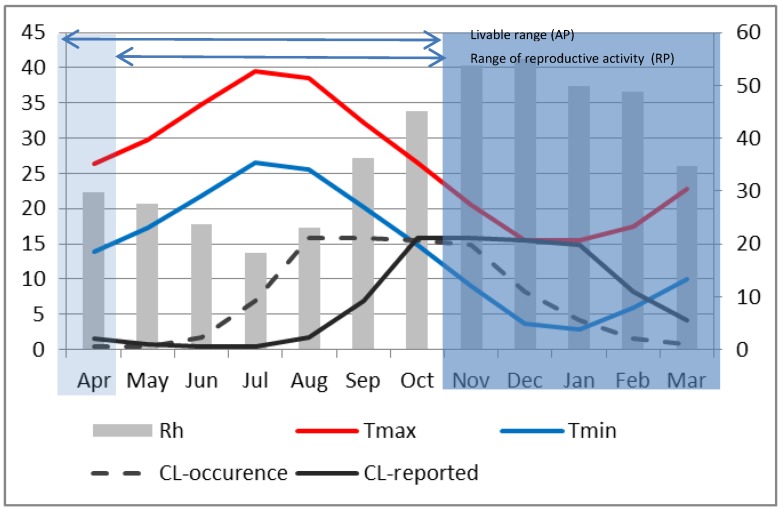
Same as Figure 6, except for Errachidia and the monthly average ZCL-cases are divided by 10 for plotting purposes.

Two main differences are apparent in Errachidia. First, unlike Saida where the seasonal maximum temperature reached only around 36 °C, it was around 40 °C in Errachidia in July and August. This is believed to have significantly reduced the sandfly activity and limited the number of ZCL-cases. Indeed the seasonal variation of reported ZCL-incidence for Errachidia during P2 (Figure 7) indicates a peak in October that remains high and almost constant until January, at which time it starts to decline. Second, the reproductive period in Errachidia starts about 1 month earlier than Saida. As such the maximum reported ZCL-incidence happens in October. Applying the 2-month lag in accordance with climatic conditions, the maximum number of bites appeared to have occurred from August through November when the minimum temperature drops below the critical 10 °C threshold. At Errachidia, it appears that warmer temperatures reduced the time for female vectors to grow from conception to reproductive adults and extended the reproductive period, resulting in an earlier explosion of sand flies in July capped by extremely high, over 30 °C average monthly maximum temperatures from July to September. 

### 4.2. Endemicity

Observations over the study region indicate that both provinces experienced periods of high ZCL incidence following a period with low incidence. In Saida, during P1 (1990–1999) observations showed one to two cases per year except for 1998 during which three cases were reported. However starting in 2000 and through 2009 (P2), the incidence increased to an annual average value of 126.3 or 99.06% of all cases compared to only 0.94% during P1 (Table 1). In Errachidia the annual average number of cases jumped from 91 in P1 to 891 or 85.5% of the total in P2. This raises the question: Why was there such an important and sustainable increase in ZCL-incidence between the two periods over the region? The analyzed data suggest this increase was associated with changes in precipitation and minimum temperature.

First, the increase in precipitation led to denser vegetation that supported a larger rodent and sandfly population. Additionally, we argue that increase in the decadal mean minimum temperature over the region extended the reproductive period of the sandfly. These environmental conditions are suitable for both rodents and sandflies to reproduce in large numbers and survive in abundance throughout the winter diapause to the following cycle. 

The relationship between the decadal shift in the number of ZCL cases in the region and climate indicators suggest that changes in climate created conditions suitable for endemicity that did not previously exist and ultimately, increased and maintained high prevalence of the ZCL during the second period in both provinces. Indeed in both provinces the sandfly reproductive period extended by one month into the fall season during P2. This extension represents an increase of 25% (Saida) and 20% (Errachidia) in the length of the sandfly reproductive period and is associated with the observed increase in the minimum temperature. A paired student T-test verified the statistical significance of the difference in minimum temperatures between P1 and P2 for the AP (April-November) at the 95% confidence level for both provinces. While these preliminary results may provide some guidance as to the functional relationship between surface climate indicators and ZCL-cases, further research is needed in this populated and data sparse region in order to have a comprehensive assessment of the impact of climate on vector-borne diseases (e.g., [46,49,50]).

### 4.3. Suppression

While an increase in temperature and precipitation, possibly associated with climate change, resulted in an increase in ZCL-incidence, our data also show that beyond critical temperatures, the incidence of ZCL in the region of Saida abruptly declines. Even though data did not show temperatures above the theoretical livable range for the vector, in laboratory experiments the number of offspring per female begins to decline precipitously above 28 °C [23]. Therefore, high temperatures during the reproductive period (RP) in one year would reduce the size of the emerging population and its overall growth potential the following year. 

**Figure 8:**
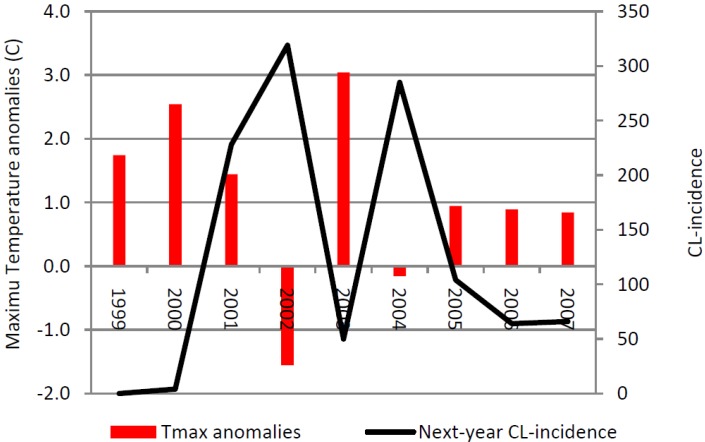
Average July-August maximum temperature anomalies from the mean value over P2 (34.76 °C) and 1-year lag ZCL-cases for Saida.

Figure 8 shows the ZCL-incidence and the annual anomaly of the maximum temperature averaged over the two hottest months (July and August) during the period of high incidence (P2) in Saida. Our analysis indicates that years with high maximum temperature anomalies are associated with a decrease in the prevalence of the disease in the following year. In Saida, ZCL-incidence displays a strong one-year lagged inverse correlation with the yearly maximum temperature anomaly explaining about 66% (r = −0.81) of the variance in ZCL-cases. This variation is marked between 2000 and 2002, where reduction of the maximum temperature by 1.10 °C between 2000 and 2001 was associated with an increase of 224 cases in 2002. Furthermore a subsequent cooling of 3.0 °C between 2001 and 2002 was associated with an increase of 91 cases in 2003 (Figure 8). On the other hand, the reduction in the number of cases is especially visible in 2004, where a significant drop in cases followed an above normal warmer year in 2003. The year 2003 experienced a positive temperature anomaly of 3.0 °C above normal, a 4.6 °C warming from the previous year, which brought the average July-August maximum temperature to about 38 °C, a value close to the upper limit of the sandfly livable range [24] and well above the 28 °C critical value limiting the number of offspring per adult female [23] which has significantly reduced the ZCL-incidence by 269 cases the following year 2004. Unlike Saida, the annual data for Errachidia does not show any significant outliers in terms of decreases in ZCL incidence. The average July-August maximum temperature over the same period in Errachidia was approximately 37.8 °C with anomalies confined between 0.25 and 2.2 °C indicating a consistently higher temperature regime during P2. 

## 5. Conclusions

*Phlebotomus papatasi* is an important vector of epidemiological consequence. In Semi-arid North Africa, cold winter temperatures have limited its range and prevented its establishment in much of the temperate regions along the Mediterranean coast. Consistent warming and moistening over the last decade appears to have changed the spatio-temporal distribution of the range of this insect. Understanding how climate affects the abundance and the seasonal dynamics of this vector is paramount to understanding and predicting the spread of the potential infectious disease it may transmit, cutaneous leishmaniasis. In the pre-Saharan North Africa, ZCL is a serious public health problem with significant social consequences, especially in women, due to the indelible scars that skin lesions leave on the faces of vulnerable patients. Furthermore, this region is home to growing indigenous populations that thrive to sustain livelihood. However, not much research has been conducted to describe the potential associations between climate and the incidence of the ZCL. 

Climate conditions affect the leishmaniasis complex components (parasite-reservoir-vector) and their ability to interact, persist and establish in new ecosystems. This study describes empirical relationships between *L. major* ZCL incidence and surface climate indicators. It presents observational evidence from data in two sites that changes in climate in semi-arid pre-Saharan North Africa may be the initial catalyst of a trophic cascade that results in a 1-year delayed response in rodent and sand flies population density and an additional 1-year lag in ZCL-incidence. These relationships could prove useful in predicting elevated risks of human contraction of ZCL, especially in the study region where vegetation density is highly sensitive to changes in seasonal precipitation. These relationships also support the importance of environmental surveillance of rodent’s population expansion following rainy seasons and its use as first indicator of ZCL epidemic risk level. Our study suggests the ZCL annual cycle is modulated by climate parameters with extreme temperatures and moisture playing important roles, especially during the period of transmission. In the study region, we propose a 2-month lag between the reported infection date and the presumed date when the infection actually occurred based on climate indicators and sandfly optimum physiological temperatures and humidity levels. While this result may be culturally dependent and thus regional in scope and significance, it does point to the urgent need of public awareness and education about the disease and its symptoms. ZCL appears to thrive in areas with environmental conditions that allow the vector to have multiple life cycles creating thus larger sandfly populations and a greater probability of contact with both infected and non-infected hosts. The decadal increase in the number of ZCL occurrence in the region suggests that changes in climate resulted in a sufficient increase in minimum temperatures that allowed the establishment of new endemic foci in regions that were not previously endemic. Our data also indicated that temperatures above a critical range seem to suppress ZCL-incidence, apparently by limiting the vector’s reproductive activity. 

In summary, our study adds to the evidence linking ZCL incidence to changes in surface climate and suggests simple associations between them that could help establish an early warning system to local populations of these remote regions. Further work must be done with more comprehensive clinical and climate data to gain a complete picture of possible dynamic relationships between ZCL incidence and changes in surface climate. However, the results presented here suggest that regional changes in surface climate and the state of vegetation may already be playing a role in establishing favorable environmental conditions for the ZCL to expand to northern territories in the pre-Saharan regions.
